# Dihydrolipoamide dehydrogenase of *Vibrio splendidus* is involved in adhesion to *Apostichopus japonicus*

**DOI:** 10.1080/21505594.2019.1682761

**Published:** 2019-10-24

**Authors:** Fa Dai, Weiwei Zhang, Qiuting Zhuang, Yina Shao, Xuelin Zhao, Zhimeng Lv, Chenghua Li

**Affiliations:** a State Key Laboratory for Quality and Safety of Agro-products, Ningbo University, Ningbo, PR China; b Laboratory for Marine Fisheries Science and Food Production Processes, Qingdao National Laboratory for Marine Science and Technology, Qingdao, PR China

**Keywords:** *Vibrio splendidus*, dihydrolipoamide dehydrogenase (DLD), adhesion

## Abstract

*Vibrio splendidus* is one of the most opportunistic marine pathogens and infects many important marine animals, including the sea cucumber *Apostichopus japonicus*. In this study, two genes named *DLD1* and *DLD2*, encoding dihydrolipoamide dehydrogenase (DLD) homologues in pathogenic *V. splendidus*, were cloned, and conditionally expressed in *Escherichia coli* BL21 (DE3). The enzymatic activities of DLD1 and DLD2 showed that they both belonged to the NADH oxidase family. Both DLD1 and DLD2 were located on the outer membrane of *V. splendidus* as detected by whole-cell ELISA. To study the adhesion function of DLD1 and DLD2, polyclonal antibodies were prepared, and antibody block assay was performed to detect the normal function of the two proteins. DLD1 and DLD2 were determined to play important roles in adhesion to different matrices and the adhesive ability of *V. splendidus* reduced more than 50% when DLD1 or DLD2 was defective.

## Introduction

The sea cucumber *Apostichopus japonicus* (Echinodermata, Holothuroidea) is a marine animal with important economic values [1] and is particularly important to the economy of North China [2]. The bacterial infectious diseases of *A. japonicus*, such as skin ulcer syndrome (SUS) [3], perioral swelling syndrome [4] and rotting edge syndrome [5], frequently occur in aquaculture. *Vibrio* sp. [6], *Pseudomonas* sp. [7] and spherical virus [8] are the main pathogens of *A. japonicus*. In particular, *Vibrio splendidus* is considered to be the major pathogen that infects *A. japonicus* [9]. However, until now, little is known about the pathogenic mechanism of *V. splendidus*, which has significantly hindered the development of specific prevention methods for *A. japonicus*.

The virulence factors of *Vibrio* sp. generally include adhesion factors, hemolysins, and extracellular products [10]. The metalloproteinase Vsm is involved in the interaction between *V. splendidus* and *A. japonicus* and contributes to the cytotoxicity effects on the *A. japonicus* coelomocyte [11–13]. Hemolysin Vshppd not only is involved in the cytotoxicity to coelomocyte but also contributes to the stimulatory effect on the immune response [14]. When expressed in the cytoplasm under the control of the CUP1 promoter, Vis was toxic to yeast, and catalytic variants lost the ability to kill the yeast host, indicating that the toxin exerts its lethality through its enzymatic activity [15]. These studies on the pathogenicity of *V. splendidus* are far from enough.

In general, adhesion is the first step of bacterial infection and bacterial adherence is a complicated process of interaction between a pathogen and its host [16,17]. However, there has been no report on the adhesion factor of *V. splendidus* and its adhesive process until now. Flagellar assembly-associated proteins, such as *flrA, flrB*, and *flrC*, have been reported to be typical adhesion factors [18]. In *Vibrio mimicus*, outer membrane protein U (OmpU) was shown to be involved in adhesion [19]. *V. splendidus* possesses the characteristics of strong hydrophobicity and high biofilm formation ability [20], which made us wonder whether it possesses adhesion factors or not, and what are the adhesion factors contributing to its pathogenicity. Till now, no adhesion factor has been reported in *V. splendidus*, so finding basic adhesion factors and exploring their ability is very important for the exploration of the pathogenicity of *V. splendidus*.

Dihydrolipoamide dehydrogenase (DLD) is present in a variety of organisms and is an oxidoreductase that is essential in energy metabolism [21]. It is a part of three α-pyruvate dehydrogenase complex species, and belongs to the flavin protein oxidoreductase family [22,23]. DLD not only performs the function of oxidoreductase but also plays an important role in bacterial pathogenesis. DLD was determined to be one of the virulence determinants in *Mycoplasma gallisepticum* by signature sequence mutagenesis [24]. *DLD*-deficient *Streptococcus pneumoniae* lost the ability to infect mice [25]. In the present study, two *DLD* genes were cloned, and their enzymatic activities were characterized. The localization of DLDs was also determined using whole cell enzyme-linked immunosorbent assay (ELISA) and the adhesive ability of DLD was explored.

## Materials and methods

### Bacterial strains, culture conditions and chemicals

*V. splendidus* was isolated from *A. japonicus* suffering from SUS in an indoor farms in Jinzhou Hatchery in May 2013, and its identity was determined using 16S rDNA sequence. Its pathogenicity to *A. japonicus* was determined in our previous study [26]. This bacterium was stored in glycerol at −80°C for further utilization. Unless otherwise stated, *V. splendidus* was cultured in modified Zobell’s 2216E medium at 28°C (tryptone, 5 g; yeast extract, 1 g; and FePO_4_, 0.01 g in 1 L aged seawater). *Escherichia coli* DH5α, S17λπ and BL21 (DE3) was cultured in Luria-Bertani (LB) medium at 37°C. Cell density was measured at 600 nm by a UV-Vis spectrophotometer (Beckman). Culture of *V. splendidus* or *E. coli* at an OD_600_ = 1.0 was corresponded to the cell density of 1.01 × 10^9^ CFU mL^−1^. Ampicillin (Ap, 100 μg mL^−1^) and kanamycin (Kn, 50 μg mL^−1^) were used in this study. Plasmid pMD19-T, Taq and Pfu DNA polymerase was Clontech purchased from Takara (China). Restriction endonucleases were purchased from New England Biolabs. 5-([4,6-dichlorotriazin-2-yl] amino) fluorescein hydrochloride (5-DTAF) was purchased from Sigma (USA). All the other chemicals used in this study were purchased from Sangon (Shanghai, China) unless otherwise stated.

### DNA manipulation and plasmid construction

The plasmid preparation, the extraction of DNA fragments from agarose gels and the purification of PCR products were performed using the respective kits from Omega Bio-Tek (GA) according to the manufacturer’s instructions. According to the genomic DNA of *V. splendidus* LGP32, we found two nucleotides sequences encoding *DLD*, and they were named *DLD1* and *DLD2*. Four primers, DLD1F:5ʹ-CGCGGATCCATGAGCAAAGAAATTAAA-3ʹ, DLD1R: 5ʹ-ACGCTCGAGTTACTTCTTCTTTTTCACTG-3ʹ, DLD2F: 5ʹ-GGATCCATGAAACAAGTCAATGTAGATGTAGC-3ʹ, and DLD2R: 5ʹ-CTCGAGTTAACAACCAGGACCACAGTCTA-3ʹ were designed according to the two nucleotide sequences. Using the two pairs of primers, *DLD1* and *DLD2* were amplified and ligated to the pMD19-T. pET-28a-DLD1 or pET28a-DLD2 was constructed by ligating *DLD1* or *DLD2* between the *BamH* I and *Xho* I sites of pET28a.

### Expression and purification of recombinant DLD

Overnight culture of *E. coli* BL21 (DE3)/pET28a-DLD1 or *E. coli* BL21 (DE3)/pET28a-DLD2 was inoculated into 100 mL LB medium with Kn and cultured at 37°C until the OD_600_ reached 0.5. Isopropyl-β-D -thiogalactopyranoside was added into the culture at a working concentration of 0.4 mM to induce the expression of *DLD* and the induction process lasted for 6 h at 28°C. Then, the cells were harvested by centrifuging at 8000 × g for 10 min. Cells were precipitated and used for the purification of recombinant protein. Recombinant His-tagged DLD1 or DLD2 was purified using Ni-NTA Sepharose column (Roche, Shanghai). The recombinant protein was determined by 12% SDS-polyacrylamide gel electrophoresis (SDS-PAGE) and the concentration of recombinant protein was measured using the BCA Protein Assay Kit (Sangon, China).

### Antibody preparation

Polyclonal antibodies against DLD1 and DLD2 were generated by immunizing 4-week-old mice according to the method described in [27]. Blood was taken from mice through the eyeball and polyclonal antibodies against DLD1 and DLD2 were stored at −80°C for further utilization.

### Enzymatic assay

The DLD activity of DLD1 and DLD2 was determined based on the method of Serrano [28] with slight modification. The DLD activity was calculated based on measurement of NADH oxidation using disulfide substrate as a substrate at 25°C. The reaction was monitored by the decrease in absorbance at 340 nm. Reaction mixtures contained 10 μM PBS (pH 7.0), 3 μM DL-lipoamide (ANPEL Laboratory Technologies, Shanghai), 2 μM NADH (Solarbio, China), and 100 μL of enzyme in a final volume of 3 mL.

### Whole cell ELISA

To determine whether DLD was located on the cell surface, a whole cell ELISA was performed according to the protocol of Aquatic Diagnostic Ltd. (UK). Polyclonal antibodies against DLD1 and DLD2 were diluted 1:400 with PBS. A total of 100 μL of mid-logarithmic phase *V. splendidus* at approximately 1.0 × 10^7^ CFU ^mL−1^ was added separately to a 96-well ELISA plate coated with 0.05% (w/v) poly-L-lysine. The well without incubation with *V. splendidus* cells was used as a negative group. There were three replicates for each sample. Then, 100 μL of 2.5% (v/v) glutaraldehyde was added to each well to fix cells for 4 h, and each well was washed three times with 300 μL of PBS (the plate was inverted on absorbent paper to avoid cross-contamination). After fixation, 300 μL of 1% (w/v) bovine serum albumin was added to block the sample for overnight, followed by three times washes. The cells were treated with 1:400 diluted anti-DLD polyclonal antibody above. The wells treated without the polyclonal antibody were used as the control group. After washing, the wells were treated with horseradish peroxidase-conjugated goat anti-mouse IgG (Bios, China). Color development was performed using the TMB Kit (Solarbio, China) and then 50 μL of hydrochloric acid (1 M) was added to terminate the reaction. The absorbance of the developed color was read at 450 nm with a UV-Vis spectrophotometer (Beckman) [29].

### Fluorescence labeling of V. splendidus

To observe the adhesive ability of *V. splendidus, V. splendidus* cells were stained with 5-DTAF (Sigma, USA) as described by [30] and visualized under a microscope. The working concentration of 5-DTAF was 0.005 mg mL^−1^ (dissolved in PBS). Five millilitres of mid-logarithmic phase *V. splendidus* (OD_600_ = 0.5) and 2 mL of the 5-DTAF solution were mixed in a dark environment at 28°C and incubated in an oscillating incubator for 1 h. Then, DTAF-labeled *V. splendidus* (*Vs*-DTAF) cells were collected by centrifugation at 8000 × g for 6 min and resuspended in PBS. The wells were repeatedly washed until the supernatant showed no color [31,32]. Finally, *Vs*-DTAF cells were observed under a fluorescence microscope (Nikon).

### Adhesion of V. splendidus to polystyrene

In this study, the efficiency of *V. splendidus* to adhere to polystyrene was evaluated by cell number count (CP) and epifluorescence microscopy methods [33]. *Vs*-DTAF and polyclonal antibodies (diluted 1:400 with PBS) were mixed at a ratio of 1:1 (v/v). Then, it was incubated in an oscillating incubator at 28°C for 2 h. In the control group, *V. splendidus* was mixed with an equal volume of PBS and incubated under the same conditions. Then, 400 μL of the incubated mixed solution was placed onto a polystyrene plate to adhere for 4 h in the dark. Then, the excess liquid was removed, and then the adhered cells were washed with PBS for 10 min. After washing, 2.5% glutaric dialdehyde (Solarbio, China) was added to the polystyrene plate for fixation. The polystyrene plate was observed under a fluorescence microscope and the elution was coated on 2216E medium after a 1.0 × 10^3^-fold dilution. The adhesion rate was calculated based on the number of single colonies that emerged on the plate.

### Coelomocyte culture

Healthy adult *A. japonicus* (weight 125 ± 15 g) were obtained from Zhangzi Island Aquaculture Company (Dalian, China) and acclimatized in 30 L of aerated natural seawater (salinity 28 psu, temperature 16°C) for 3 days. Primary coelomocytes were prepared according to our previous work [34]. Briefly, the harvested cells were resuspended in L-15-S cell culture medium with sodium (Invitrogen, USA), at a final concentration of 1.0 × 10^6^ cells mL^−1^. NaCl solution was utilized at a final concentration of 0.39 M to adjust the osmotic pressure. The cells were then dispensed into a 24-well culture microplate with 500 μL of L-15-S medium with sodium in each well. All the experiments were performed in 48 h to ensure that the cells were in a healthy adherent state.

### Adhesion of V. splendidus to coelomocytes

According to the cell adhesion counting method of [35], coelomocytes were cultured in a 24-well plate. A total of 0.5 mL of L-15-S cell culture medium with sodium (Invitrogen, USA) was added to each well until the monolayer cells were cultured. The wells were divided into three groups: one group was incubated with *Vs*-DTAF preincubated with polyclonal DLD1 antibody. The second group was incubated with *Vs*-DTAF preincubated with polyclonal DLD2 antibody. The control group was incubated with *V. splendidus*. The antibody was preincubated with *Vs*-DTAF at a ratio of 1:1 (v/v), with three replicates for each group. After incubation for 2 h, the spent culture medium was removed and 400 μL of the mixtures was added to each well. The 24-well plate was incubated at room temperature for 3 h. After that, the suspension was removed and the cells that adhered to the coelomocytes were resuspended in 1 mL of PBS. The resuspension was 1.0 × 10^1^- to 1.0 × 10^4^-fold diluted and plated on 2216E medium. The rest of the resuspension was stained with Dil (Beyotime, China) and visualized using a laser scanning spectral con-focal microscope (TCS SP2; Leica, Solms, Germany).

### Adhesion of V. splendidus to tissues of A. japonicus

Ten individuals of healthy adult *A. japonicus* (weight 125 ± 15 g) were obtained from Zhangzi Island Aquaculture Company (Dalian, China) and acclimatized in 30 L of aerated natural seawater (salinity 28 psu, temperature 16°C) for 3 days. We divided the *A. japonicus* individuals into three groups in 10 L of aerated natural seawater, and there were three *A. japonicus* specimens for each group. A total of 0.1 L *V. splendidus* (OD_600_ = 1.0) and polyclonal antibodies (diluted 1:400 with PBS) were mixed at 28°C and incubated in an oscillating incubator for 2 h at a ratio of 1:1 (v/v) and then centrifuged at 8,000 g for 10 min. The control group was *V. splendidus* (0.1 L, OD_600_ = 1.0) mixed with the equivalent amount of PBS. After immersion infection for 24 h, the body wall, tentacle, muscle, respiratory tree and intestine of *A. japonicus* were collected and weighed. For each sample, three *A. japonicus* specimens were collected. Tissue samples were homogenized by a homogenizer with sterilized PBS and then the homogenate was 1.0 × 10^1^- to 1.0 × 10^4^-fold diluted and plated on 2216E medium. The plates were cultured at 28°C for 24 h [36,37].

### Sequence analysis

The sequences of *DLD1* and *DLD2* were analyzed using the BLAST algorithm at the National Centre for Biotechnology Information (http://www.ncbi.nlm.nih.gov/blast). The deduced amino acid sequences were analyzed using the Expert Protein Analysis System (http://www.expasy.org/). The molecular mass (MM) and theoretical isoelectric point (*pI*) were calculated by the Protparam tool (http://www.expasy.ch/tools/protparam.html). The putative signal peptide cleavage site was identified using the SignalP 4.1 Server (http://www.cbs.dtu.dk/services/SignalP/). Domain in these amino acid sequences was detected using the simple modular architecture research tool (SMART) program (http://www.smart.emblheidelbergde/) and multiple alignment analysis of proteins was performed using the Clustal Omega Multiple Alignment program (http://www.ebi.ac.uk/clustalw/) [38]. The neighbor-joining phylogenetic tree was constructed using Mega 5.0 program with 1000 bootstraps [39].

## Results

### Cloning of DLD and sequence analysis of DLD

**Figure 1.**
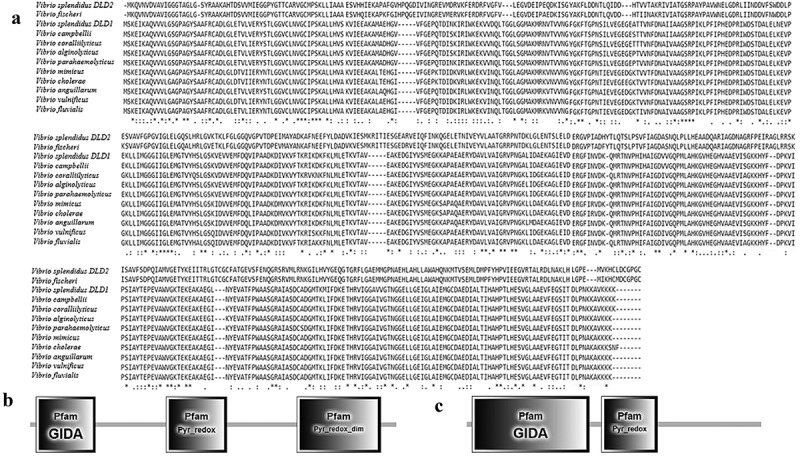
(a): Multiple sequence alignment of DLDs from *Vibrio* spp. The GenBank accession numbers are as follows: DLDs of *Vibrio fischeri* WP_069594165.1, *Vibrio splendidus* (DLD1) WP_010435969.1, *Vibrio splendidus* (DLD2) WP_004729673.1, *Vibrio campbellii* WP_012128871.1, *Vibrio coralliilyticus* WP_006958123.1, *Vibrio parahaemolyticus* WP_021823119.1, *Vibrio alginolyticus* AGK62253.1, *Vibrio mimicus* WP_000031532.1, *Vibrio cholerae* WP_000031535.1, *Vibrio anguillarum* WP_013857673.1, *Vibrio vulnificus* RAH35251.1, *Vibrio fluvialis* WP_020430460.1. (b and c) showed the domains of DLD1 and DLD2.

**Figure 2.**
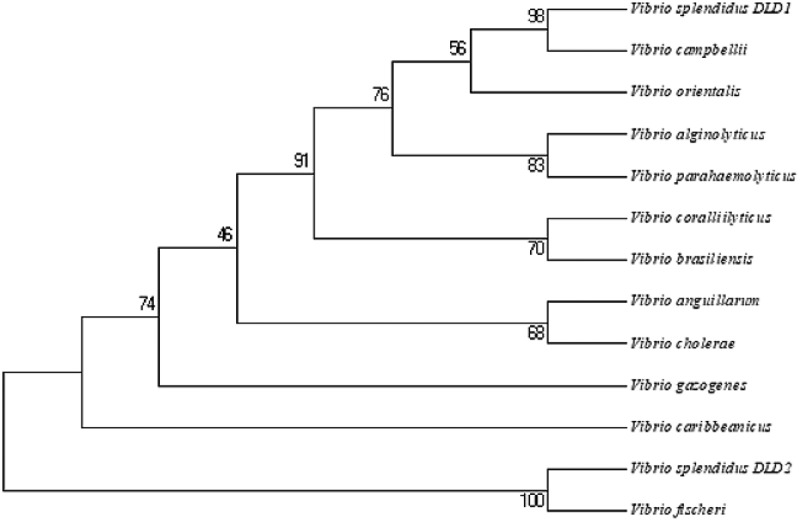
Unrooted phylogenetic tree of DLDs from different *Vibrio* spp. constructed using MEGA 5.0 software. The scale bar represents a distance of 0.1 substitutions per site. The tree was obtained by bootstrap analysis with a neighbor-joining method, and numbers on branches represent bootstrap values for 1000 replications.

There were two proteins annotated as DLD, named DLD1 (WP_010435969.1) and DLD2 (WP_004729673.1) from the whole sequence of *V. splendidus* LGP32. *DLD1* was amplified by PCR with the primers DLD1F and DLD1R. Nucleotide sequence analysis showed that the ORF of *DLD1* was 1431 bp. *DLD1* encoded a protein with an estimated molecular mass of 50.9 kDa, and its theoretical *pI* was 5.62. Likewise, *DLD2* was amplified by PCR with the primers DLD2F and DLD2R. Nucleotide sequence analysis showed that the ORF of *DLD2* was 1467 bp. *DLD2* encoded a protein with an estimated molecular mass of 53.16 kDa, and its theoretical *pI* was 5.28. SingalP prediction revealed that both *DLD1* and *DLD2* had no typical signal peptide. BLAST showed that the amino acid sequences of DLD1 and DLD2 were similar to those of *Vibrio alginolyticus, Vibrio crassostreae*, and *Vibrio fischeri*, and the similarity reached 99%. Multiple sequence alignment showed a highly conserved amino acid sequence between *DLD1* and other *Vibrio* spp., while *DLD2* showed a slightly lower similarity (Figure 1(a)). Smart analysis showed that both DLD1 and DLD2 contained a Pyr_redox domain and GIDA domain (Figure 1(b,c)). They both showed close evolutionary relationships with other *Vibrio* spp. in the phylogenetic tree analysis (Figure 2).

### Purification of recombinant DLD and enzymatic activity

**Figure 3.**
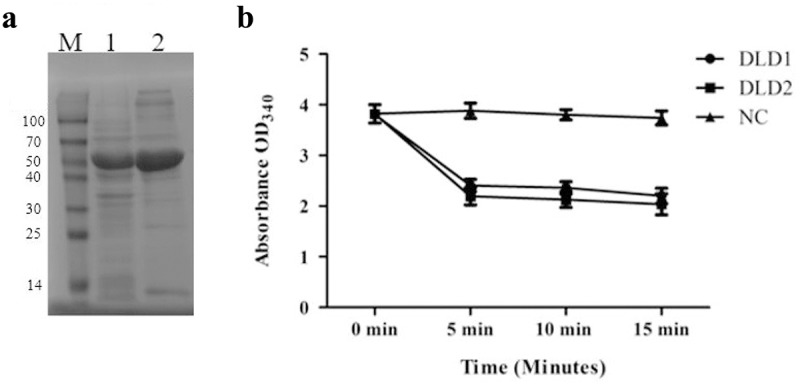
(a): the SDS-PAGE analysis of the recombinant proteins of DLD1 (lane 1) and DLD2 (lane 2), and M is the Marker (kDa). (b): the absorbance at OD_340_ after the reaction was catalyzed by DLD1 or DLD2. The reaction without DLD1 or DLD2 was used as a control (NC). Data are the means of three independent experiments, and are presented as means ± SD.

The recombinant DLD was expressed in *E. coli* BL21 cells, and purified using Ni-NTA Sepharose column. The recombinant proteins are shown in Figure 3(a). Before the addition of DLD1 or DLD2, the NADH solution was clear yellow. After the addition of the recombinant protein, the color of the mixture turned from yellow to colorless. The absorbance dropped rapidly within the first 5 min of the reaction and the solution tended to be colorless, with an OD_340_ decrease from 3.8 to 2.06 or 2.12 in the presence of DLD1 or DLD2, respectively. However, there were no obvious changes in the control group (Figure 3(b)).

### Localization of DLD in V. splendidus

**Figure 4.**
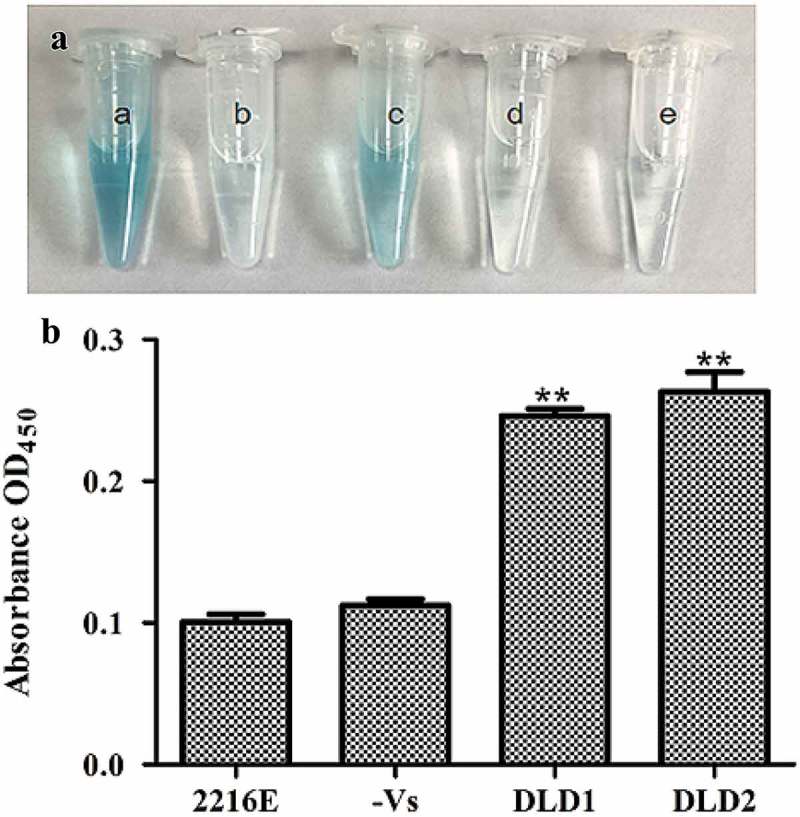
Determination of DLDs location using whole cell ELISA. The same amount of cells was used as antigen to react with DLD1 or DLD antibody 2. The developed color was measured at 450 nm. (a): a, the cells blocked by DLD1 antibody; b, the negative group; c, the cells blocked by DLD2 antibody; d, the negative group; e, the control group. (b): The absorbance at OD_450_ of the control group, negative group, *V. splendidus* reacted with DLD1 antibody and *V. splendidus* reacted with DLD2 antibody. Data are the means of three independent experiments, and are presented as means ± SD. *P< 0.05, **P< 0.01.

We detected if DLDs were located on the cell surface of *V. splendidus* using whole cell ELISA. After the whole cell reacted with an antibody against DLD1 or DLD2, the color of the experimental group changed to blue, while there was no significant color change in the control group (no antibody) or negative group (no *V. splendidus*). The absorbance at OD_450_ of cells that reacted with DLD1 or DLD2 was 0.25 (Figure 4(b)) or 0.27 (Figure 4(c)), respectively, which was obviously higher compared with the control group. All the obtained results confirmed the fact that DLD1 and DLD2 are located on the cell surface of *V. splendidus*.

### Adhesion of V. splendidus to polystyrene

**Figure 5.**
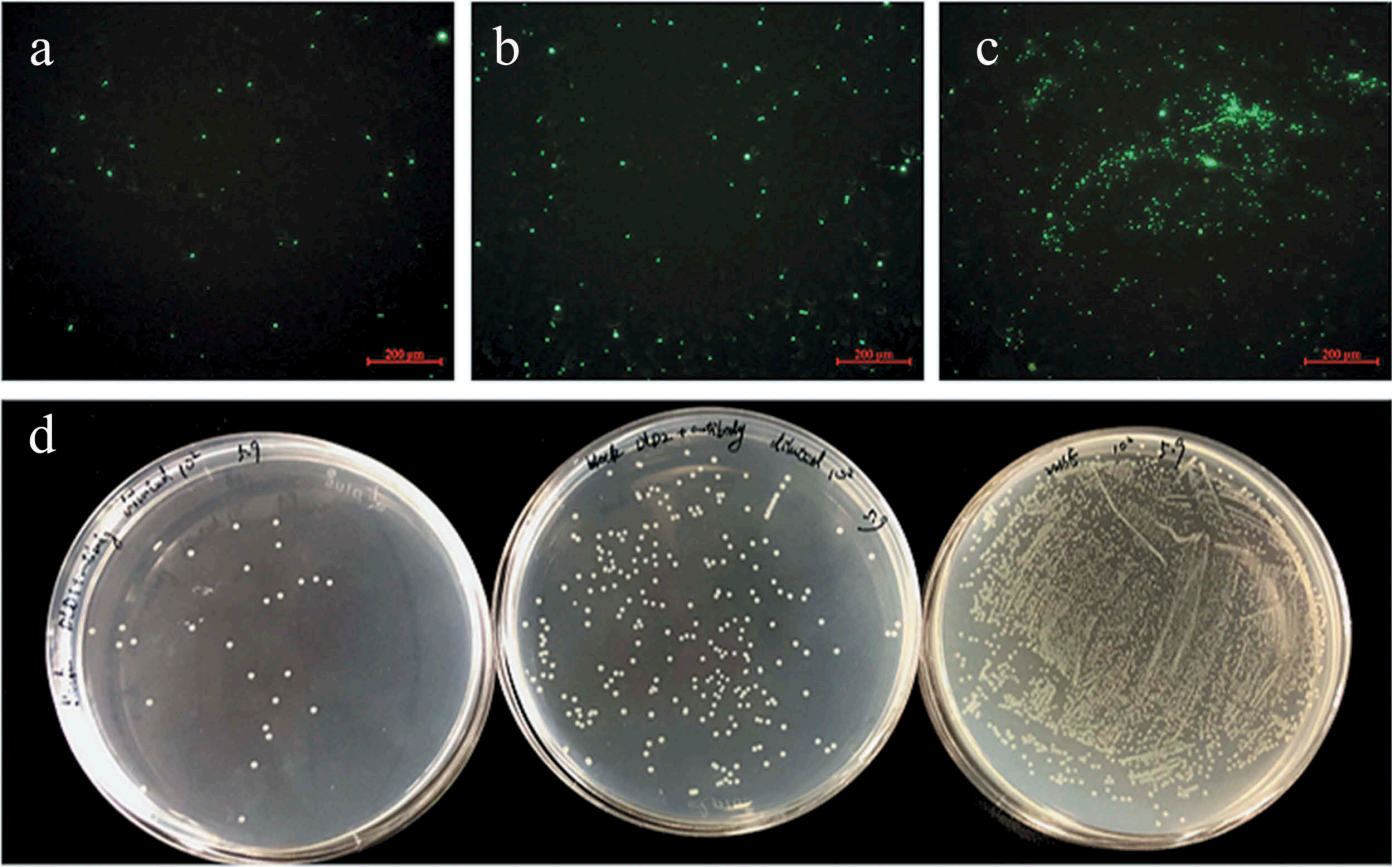
The adhesive ability of *V. splendidus* to polystyrene demonstrated by fluorescence (a-c) and colony counting (d). The green fluorescence was generated by the labeled *Vs*-DTAF. a, the cells blocked by DLD1 antibody; b, the cells blocked by DLD2 antibody; c, the control group; d, quantity of the cells that adhered to polystyrene. From the left to right: *V. splendidus* treated with DLD1 antibody, *V. splendidus* treated with DLD2 antibody, and *V. splendidus*.

For the adhesion assay, *Vs*-DTAF at a concentration of 8.0 × 10^7^ CFU mL^−1^ was incubated with antibodies of either DLD1 or DLD2. The cells that adhered to the polystyrene were significantly reduced after incubation with the DLD1 or DLD2 antibody. Under the fluorescence microscope, the number of luminous *V. splendidus* cells preincubated with polyclonal DLD1 or DLD2 antibody was obviously less than that of the control group (Figure 5(a–c)). Moreover, the cells blocked by the DLD1 antibody were slightly less adhesive than that of the cells blocked by the DLD2 antibody. Quantitative determination of the amount of the adhered bacteria was performed by the viable cell counting method (Figure 5(d)). The adhesive rate to the polystyrene of *V. splendidus* without antibody blocking was approximately 1%, but after blocking with DLD1 or DLD2 antibody, the adhesive rate decreased to 0.149% or 0.168%, respectively.

### Adhesion of V. splendidus to coelomocytes

**Figure 6.**
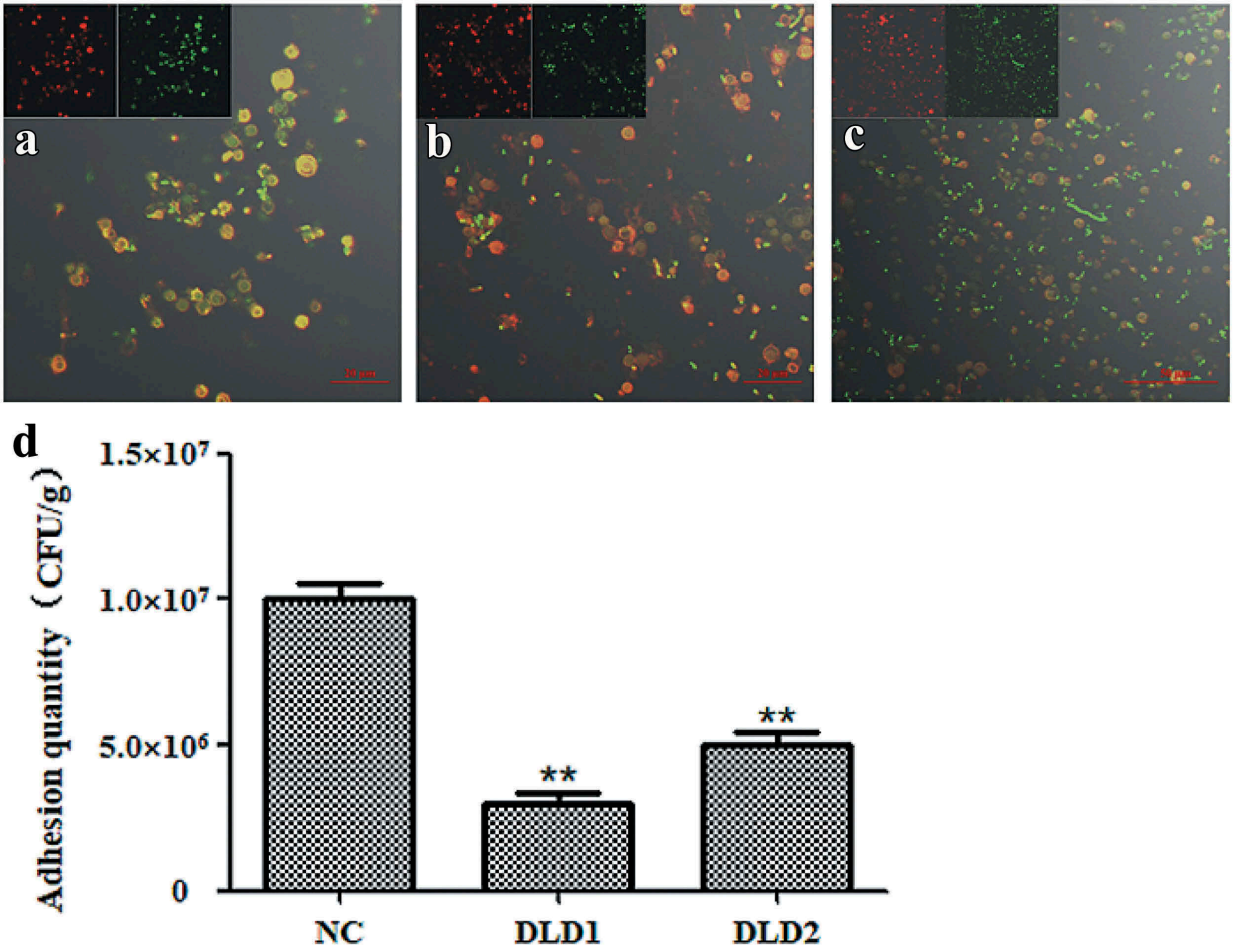
The adhesive ability of *V. splendidus* to coelomocytes demonstrated by fluorescence (a-c) and colony counting (b). The green fluorescence was the labeled *Vs*-DTAF and the red fluorescence was the labeled coelomocyte. (a), the cells blocked by DLD1 antibody; (b), the cells blocked by DLD2 antibody; (c), the control group. (d), Colony counting of the cells that adhered to coelomocytes. After adhesion and washes, the resuspesion cell solution was 1.0 × 10^3^-fold dilution, and 50 μL was spread on 2216E plate. Data were means of three independent experiments, and are presented as means ± SD. *P< 0.05, **P< 0.01.

We further tested the adhesive ability of *V. splendidus* to coelomocytes after blocked with DLD1 or DLD2 antibody. Under the laser scanning spectral confocal microscope, the adhesive *Vs*-DTAF after antibody blocking (Figure 6(a,b)) was obviously less than that of the control group (Figure 6(c)). Based on the number of bacteria that adhered to coelomocytes showed that after blocking with DLD1 or DLD2 antibody, the adhesion quantity was significantly reduced, and the number of cells blocked with the DLD1 antibody was reduced more than that of cells blocked with the DLD2 antibody (Figure 6(d)). The adhesive rate of wide-type *V. splendidus* to the coelomocytes was 25% in the control group, while the adhesive rate of the cells blocked by the DLD1 antibody was 7.5% and the adhesive rate of the cells blocked by the DLD2 antibody was 12.5%. Thus, *V. splendidus* also showed adhesive ability to coelomocytes of *A. japonicus*, and DLD1 and DLD2 were also involved in the adhesion of *V. splendidus* to coelomocytes of *A. japonicus*.

### Adhesion of V. splendidus to tissues of A. japonicus

**Figure 7.**
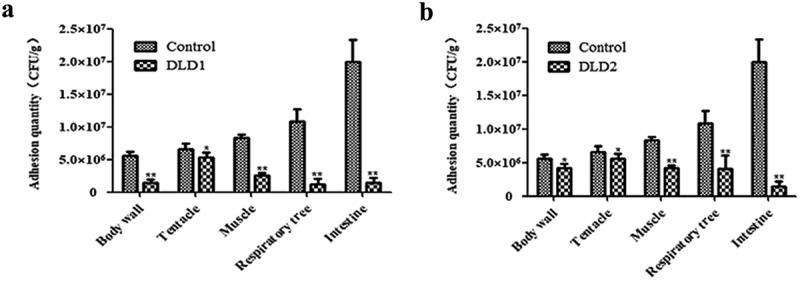
The adhesive ability of *V. splendidus* to different tissues of *A. japonicus*. The tissues of body wall, tentacle, muscle, respiratory tree, and intestine of *A. japonicus* specimens were collected and homogenized after 24 h immersion infection. The homogenate was 1.0 × 10^3^-fold diluted and 50 μL was spread on 2216E plate. (a): the cells blocked by DLD1 antibody; (b): the cells blocked by DLD2 antibody; Data are means of three independent experiments, and are presented as means ± SD. *P < 0.05, **P< 0.01.

After immersion infection, the individual *A. japonicus* specimens started to shrink and become soft compared with the individuals in the control group. The color of the respiratory tree and intestine became noticeably darker. The quantitative detection of cell adhesion quantity is shown in Figure 7. The adhesion quantities of the control group to the tissues of body wall, tentacle, muscle, respiratory tree and intestine were 5.57 × 10^6^ CFU g^−1^, 6.54 × 10^6^ CFU g^−1^, 8.39 × 10^6^ CFU g^−1^, 1.07 × 10^7^ CFU g^−1^ and 1.92 × 10^7^ CFU g^−1^, respectively. Expect for the tentacle, the adhesion quantity of *V. splendidus* treated with antibodies to other tissues was obviously decreased. DLD1 and DLD2 showed different adhesive abilities to the body wall of *A. japonicus*, in which DLD1 showed a stronger adhesive ability than DLD2.

## Discussion

In this study, full-length sequences of *DLD1* and *DLD2* in *V. splendidus* were amplified. It was found that DLD was widely present in *Vibrio* spp. with high conservation and the homology similarity of DLD1 and DLD2 to the DLDs from other *Vibrio* spp. reached 98%-100%. SingalP prediction revealed that both DLD1 and DLD2 had no typical signal peptide. Similar to glyceraldehyde-3-phosphate dehydrogenase in *Lactobacillus reuteri* [40], DLDs are also located on the cell surface of *V. splendidus* without a typical signal peptide. Smart analysis showed that both DLD1 and DLD2 contained a Pyr_redox domain and GIDA domain. The GIDA domain is a tRNA modification enzyme that is found in bacteria and mitochondria and its precise molecular function is unknown [41]. The Pyr_redox domain family includes both class I and class II oxidoreductases as well as NADH oxidases and peroxidases. This domain is actually a small NADH-binding domain within a larger FAD-binding domain [42]. After analysis of the domain in DLDs from other bacteria, it was determined that the GIDA domain and Pyr_redox domain were ubiquitously present in DLDs. Thus, we found that the GIDA domain and Pyr_redox domain are probably the main functional domains of DLD. Unlike other bacteria, there were two genes coding DLDs in *V. splendidus*, and both DLD1 and DLD2 belonged to NADH oxidases.

DLD is a multiple enzyme that is ubiquitously present in microbes, plants, and animals and plays an important role in energy metabolism. Altered energy metabolism, including reductions in the activities of the key mitochondrial enzymes alpha-ketoglutarate dehydrogenase complex (KGDHC) and pyruvate dehydrogenase complex (PDHC), are characteristic of many neurodegenerative disorders including Alzheimer’s disease, Parkinson’s disease and Huntington’s disease. DLD is a critical subunit of KGDHC and PDHC [43]. A previous study concluded that DLD is a multifunctional molecule. DLD plays a role not only in growth but also in biofilm formation. DLD is involved in drug resistance, and the expression of DLD differs among different wild type and drug-resistant strains of *V. parahaemolyticus* [44]. DLD is also involved in biofilm formation in *V. alginolyticus* and the *DLD*-deficient strain was attenuated in swimming and significantly reduced in biofilm formation [45]. Our study was the first to explore the function of DLD in *V. splendidus*. The two DLDs showed obvious adhesive abilities on various matrices, from polystyrene to host (coelomocytes and tissues). Additionally, the binding of adhesion factors to the host has strong tissue specificity in *E. coli* [46]. In our present study, it seemed that *V. splendidus* adherence to the intestine was stronger than that in the other tissues, and both DLD and DLD2 showed the smallest ability to adhere to tentacles. Although DLD1 is similar to DLD2 both in enzymatic activity and adhesive ability, DLD2 showed weaker adhesive ability than DLD1, especially in the body wall.
